# Peripheral metabolism of lipoprotein-amyloid beta as a risk factor for Alzheimer’s disease: potential interactive effects of *APOE* genotype with dietary fats

**DOI:** 10.1186/s12263-023-00722-5

**Published:** 2023-02-25

**Authors:** Zachary J. D’Alonzo, Virginie Lam, Ryu Takechi, Michael Nesbit, Mauro Vaccarezza, John C. L. Mamo

**Affiliations:** 1 Faculty of Health Sciences, Curtin Medical School, Curtin University, Perth, Western Australia Australia; 2 Faculty of Health Sciences, Curtin Health Innovation Research Institute, Curtin University, Perth, Western Australia Australia; 3 Faculty of Health Sciences, School of Population Health, Curtin University, Perth, Western Australia Australia

**Keywords:** Amyloid-beta, Lipoprotein, Vascular, Saturated fat, Nutrition, Dementia, Genetic, APOE, Alzheimer’s disease

## Abstract

Alzheimer’s disease (AD) is a progressive neurodegenerative disorder pathologically characterized by brain parenchymal abundance of amyloid-beta (Aβ) and the accumulation of lipofuscin material that is rich in neutral lipids. However, the mechanisms for aetiology of AD are presently not established. There is increasing evidence that metabolism of lipoprotein-Aβ in blood is associated with AD risk, via a microvascular axis that features breakdown of the blood-brain barrier, extravasation of lipoprotein-Aβ to brain parenchyme and thereafter heightened inflammation. A peripheral lipoprotein-Aβ/capillary axis for AD reconciles alternate hypotheses for a vascular, or amyloid origin of disease, with amyloidosis being probably consequential. Dietary fats may markedly influence the plasma abundance of lipoprotein-Aβ and by extension AD risk. Similarly, apolipoprotein E (Apo E) serves as the primary ligand by which lipoproteins are cleared from plasma via high-affinity receptors, for binding to extracellular matrices and thereafter for uptake of lipoprotein-Aβ via resident inflammatory cells. The epsilon *APOE ε4* isoform, a major risk factor for AD, is associated with delayed catabolism of lipoproteins and by extension may increase AD risk due to increased exposure to circulating lipoprotein-Aβ and microvascular corruption.

## Statement of significance

This review critically analyses an alternative pathway of AD known as the ‘vascular hypothesis’ and, for the first time, will identify SFA and *APOE* genotype as risk factors for AD through their putative roles in increase plasma L-sAβ.

## Introduction

Alzheimer’s disease (AD) is a progressive neurodegenerative disorder that accounts for approximately 70% of dementia and presently affecting in excess of 35 million [1]. Global prevalence of AD is increasing and is strongly associated with ageing [2]. There is also accumulating evidence that lifestyle choices including exercise, sleep and particularly diet are associated with heightened AD risk [3–9].

An equivocal diagnosis of AD is based on later-in-disease evidence of cerebral toxic lipofuscin aggregates within the central nervous system (senile plaque) that are enriched in the protein amyloid-beta (Aβ) neutral lipids and metals [10, 11]. However, microvascular disturbances and cognitive impairment are ordinarily indicated many years preceding frank amyloidosis and neutral lipid aggregation [12, 13], suggesting that a ‘soluble’-amyloid-microvascular-lipid axis triggers the onset and progression of sporadic AD.

An increasing number of studies report that blood measures of soluble-Aβ in cognitively healthy subjects can predict risk for AD at later age [14–19]. Specifically, the relative abundance of the Aβ_1-42_ relative to the Aβ_1-40_ isoform is reported by several laboratories to be a sensitive surrogate marker of AD risk in later life. Fandos et al. reported that in cognitively normal individuals, Aβ_1-42_/Aβ_1-40_ was associated with higher levels of cerebral Aβ plaques [20]. Plasma Aβ biomarkers were also described by Rembach et al. in predicting AD association, with baseline Aβ_1-42_/Aβ_1-40_ ratios associated with future cognitive decline [19].

Differences in the concentration of Aβ isoforms in blood may reflect changes in brain efflux of Aβ from cerebrospinal fluid to blood and therefore be indicative of central changes in amyloid metabolism. However, there is an accumulating body of evidence that plasma Aβ principally reflects changes in peripheral synthesis, secretion and metabolism of Aβ as an apoprotein of lipoproteins that are secreted from peripheral lipogenic organs [21–28].

This focussed review article considers contemporary evidence supporting the hypothesis that AD is associated specifically with aberrant peripheral metabolism of lipoprotein-Aβ. Herein, we extend the hypothesis to consider putative interactive effects of *APOE* genotype with specific dietary fats for onset and progression of AD.

### Peripheral metabolism of amyloid-beta

**Fig. 1:**
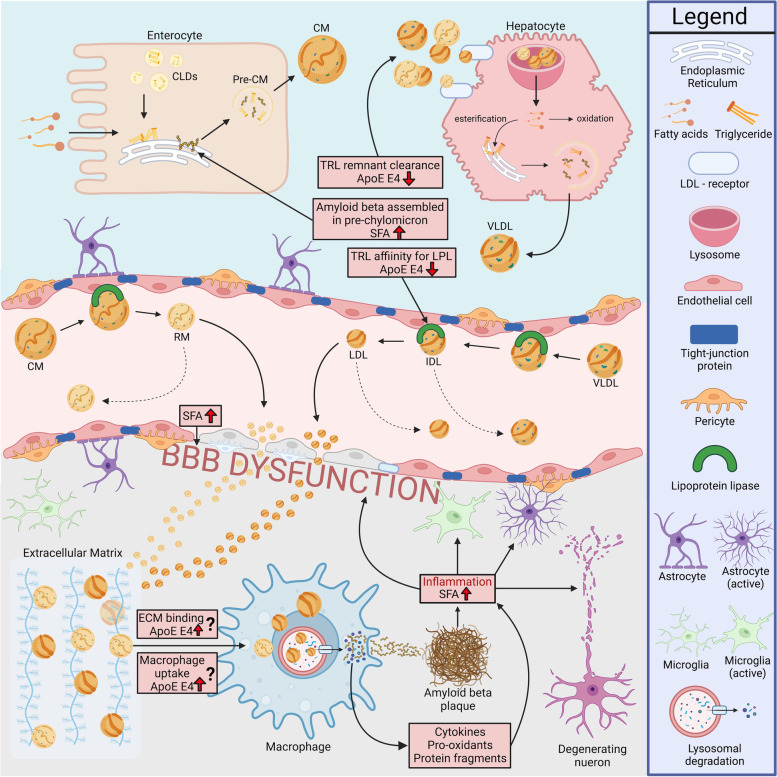
Proposed plasma lipoprotein-amyloid effects on the neurovascular unit. Dietary fats are absorbed as nonesterified fatty acids on the apical membrane of duodenal enterocytes, re-sterified and transiently stored as cytoplasmic lipid droplets. Chylomicron assembly is continuous but can be stimulated by accumulation of enterocytic lipids. Nascent chylomicrons (CM) are secreted into lymphatics with apoproteins, including amyloid-beta (Aβ), which regulate CM metabolism. In circulation, triglyceride-rich CM are progressively hydrolyzed by endothelial lipoprotein lipase, abundant on the plasma membrane of capillary endothelia. Triglyceride-depleted CM remnants (RM) are cleared from blood via an ApoE-dependent high-affinity processes, principally via the LDL receptor which is expressed in abundance on liver hepatocytes. The residual delivery of CM lipids stimulates genesis of nascent very-low-density lipoproteins (VLDL), which like CM are rich in triglyceride and share the same metabolic pathway of lipase-mediated hydrolysis and remnant clearance. VLDL-Aβ secretion may also be exaggerated because of genetic or endocrine-based comorbidities. Exaggerated abundance of lipoprotein-Aβ (CM-Aβ & VLDL-Aβ) is associated with capillary dysfunction, including attenuation of tight junction proteins and extravazation to brain parenchyme of the lipoprotein-amyloid. ApoE anchors the Aβ containing lipoprotein remnants to extracellular matrices and thereafter for receptor and phagocytic uptake of remnant lipoproteins by glia and monocyte-derived macrophages. Lysosomal degradation within the macrophage results in lipoprotein breakdown and protein hydrolylsis concomitant with the release of inflammatory cytokines and prooxidants. Focal inflammation and oxidative stress compromises neuronal cell integrity. Amyloid liberation within the cell and/or following macrophage cell death heightens propensity for Aβ aggregation. Diets enriched in saturated fatty acids may drive lipoprotein-Aβ synthesis and secretion, compromise the capillary endothelia and increase endoplasmic reticulum and mitochondrial stress. ApoE E4 relative to ApoE E3 may slow hydrolysis of triglyceride lipolysis and have lower affinity for hepatic receptor-mediated clearance pathways. Increased ApoE E4-mediated capillary exposure to lipoprotein-Aβ exacerbates capillary stress. ApoE E4 may regulate binding to matrices or regulate phagocytic uptake by inflammatory cells

Greater than 90% of blood Aβ1-40 and 97% of blood Aβ1-42 are associated with plasma lipoproteins [29]. Liver hepatocytes and small intestinal enterocytes (the site of dietary fat absorption) secrete Aβ associated with nascent very-low-density lipoproteins (VLDL) and chylomicrons, respectively [21, 30] (Fig. 1). Aβ is suggested to serve as a regulating apoprotein of triglyceride-rich lipoproteins (TRL), although few studies have investigated this directly. Consistent with the latter, in animal models, dietary-fat-induced lipogenesis was found to stimulate Aβ synthesis and secretion into blood and in other studies; obesity was reported to be positively associated with circulating Aβ and increased expression of amyloid precursor protein in adipocytes [31–33].

There is a paucity of studies that have investigated plasma lipoprotein-Aβ homeostasis in the context of AD risk per se. In a relatively small study, it was reported that patients with AD had greater net abundance of lipoprotein-Aβ in blood compared to aged-matched controls, particularly indicated within the triglyceride-rich fraction of plasma lipoproteins [34]. Moreover, the authors reported that in response to a dietary fat challenge, the AD subjects had a fourfold exaggerated post-prandial chylomicron response, compared to aged-matched controls. Additionally, several studies have reported dietary affects on plasma Aβ levels [35–37]; therefore, repeated cycles of potentially exaggerated postprandial hyperamyloidemia generated through diet could notionally accelerate lipoprotein-Aβ-induced breakdown and inflammation of the neurovascular unit (Fig. 1).

### Nutrition and Alzheimer’s disease

There is a substantial body of evidence through population; clinical and preclinical studies that demonstrate nutritional status influence risk for and progression of Alzheimer’s disease. Broad mechanisms may include modulation of neurovascular inflammation [7], through epigenetic factors and neurotransmitter modulation [38]. Nutritional homeostasis is critical for cell metabolism and bioenergetics, and indirect modulation of diet and AD risk includes maintaining a healthy body weight and reducing vascular risk factors [39]. Contemporary studies also suggest a gut/brain axis via dietary modulation of the gut microbiota [40].

### Saturated fatty acids and Alzheimer’s disease risk

Diets enriched in fat have been associated with heightened AD risk [41, 42]. Previous longitudinal studies have identified that individuals with a higher intake of saturated fatty acids (SFA) relative to unsaturated fatty acids had an increased risk of developing mild-cognitive impairment (MCI) and AD later in life [43–46]. The cardiovascular risk factors, ageing and dementia study aimed to identify a link between dietary fat consumption at midlife and subsequent effects on cognitive function. Eskelinen et al. discovered after a mean follow-up time of 21 years that chronic consumption of SFA-rich foods was associated with a reduction in global cognition, prospective memory and an increased risk of MCI [43]. Moreover, a recent meta-analysis of prospective cohort studies identified a link between SFA and AD, with individuals chronically consuming a diet richer in SFA having a greater risk of developing AD [47].

To investigate potential mechanisms underlying a putative link between dietary SFA, the peripheral metabolism of lipoprotein-Aβ and risk for AD, in preclinical studies, wild-type mice were randomized to chronically consume diets enriched in either saturated, polyunsaturated or unsaturated triglyceride. It was found that mice ingesting the SFA-rich diet had significantly increased Aβ abundance colocalized with apolipoprotein (Apo) B within the perinuclear region of enterocytes, the site of chylomicron synthesis [30]. Apo B is an obligatory protein necessary for secretion of nascent TRL from hepatocytes and enterocytes. Exaggerated chylomicron-Aβ secretion in SFA fed mice was also associated with breakdown of the blood-brain barrier (BBB), resulting in brain parenchymal extravasation of plasma proteins including plasma-derived lipoprotein-amyloid, neurovascular inflammation, neuronal degeneration and cognitive impairment, indicated by activation of astrocytes and microglia [48] (Fig. 1). In contrast, mice randomized to a diet enriched in unsaturated, or polyunsaturated fatty acids, had no evidence of increased lipoprotein-Aβ genesis and with preservation of cerebral capillary integrity and cognitive function. The differential effects of nonesterified fatty acids in the wild-type mice provide insight as to the potential mechansism underlying the association of SFA with AD reported in population studies.

### Lipoprotein-amyloid beta metabolism and Alzheimer’s disease

**Fig. 2:**
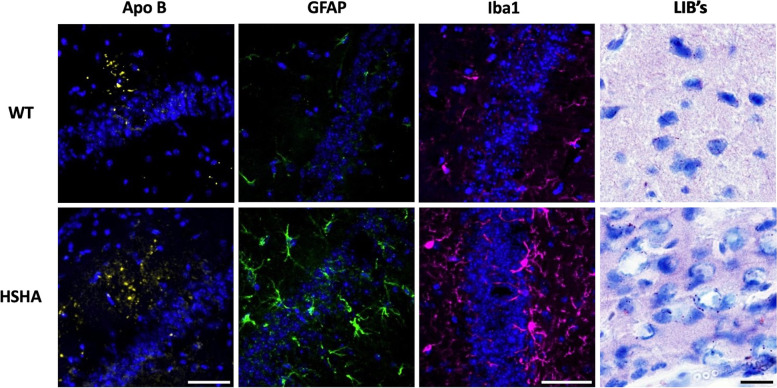
Three-dimensional confocal immunomicrographs of Apo B, GFAP, Iba1 and lipids. Apo B (yellow), activated astrocytes (GFAP; green) and activated microglia (Iba1; magenta) were all measured in the hippocampus of wild-type (WT) control mice and hepatic-specific-human amyloid (HSHA) mice (cell nuclei indicated in blue), with lipid inclusion bodies (LIB’s) measured in the cerebral cortex. Apolipoprotein (Apo) B, an exclusive marker of hepatic and intestinally derived lipoproteins, was measured at 8 months whilst glial fibrillar acidic protein (GFAP) GFAP, ionized calcium-binding adaptor molecule 1(Iba1) and lipids were all measured at 6 months. Apo B, GFAP and Iba1 all use same scale; white scale bar = 50 μm. LIBs of the cortex; black scale bar = 20 μm). WT, wild type; HSHA, hepatocyte-specific human amyloid; Apo B, apolipoprotein B; GFAP, glial fibrillary acidic protein; Iba1, ionized calcium-binding adaptor molecule 1; LIBs, lipid-inclusion bodies

Strong evidence of a lipoprotein-Aβ cascade hypothesis for cerebral capillary corruption and cognitive decline was very recently demonstrated in C57BL/6J mice that were genetically engineered to secrete exclusively from the liver, human-Aß (hAß) as an apoprotein of nascent VLDL [21]. The liver-specific amyloid transgenic mice had VLDL-hAβ at concentrations in blood that were physiologically relevant but nonetheless showed marked neurovascular inflammation and astrogliosis (Fig. 2) and cerebral accumulation of amyloid compared to control mice. Liver-specific amyloid transgenic mice also had accelerated evolution of otherwise naturally occurring, but potentially cytotoxic, age-associated lipid-inclusion bodies (Fig. 2), particularly within the hippocampal formation, a region critical to episodic memory. The liver-specific amyloid transgenic mice had chronically exaggerated rates of neurodegeneration across their lifespan, resulting in premature and significant cognitive decline compared to aged-matched controls.

Clinical evidence of a peripheral lipoprotein-Aβ hypothesis as a risk factor for AD is supported in post-mortem analysis of AD patient brains. Immunohistochemical staining of Apo B, which is synthesized exlusively by hepatocytes and enterocytes, was clearly indicated within senile Aβ-rich plaques, consistent with causality [49].

### Apolipoprotein E4 and synergistic effects with SFA may influence the metabolism of circulating L-Aβ

In humans, ApoE exists in 3 isoforms, E2, E3 and E4, with a frequency of 7%, 78% and 15%, respectively [50]. ApoE E4 is an established risk factor for AD, increasing risk for early onset by 2.8 and 8-fold for hetero- and homozygosity respectively [51, 52]. The mechanisms underlying the *APOE ε4* association are not yet established. However, evidence of interactive effects of *APOE* genotype with diet is suggested by findings that younger *APOE ε4* carriers in preclinical stages may benefit mostly from lifestyle interventions, whereas older *APOE ε4* noncarriers with dementia may show the most pronounced effects [53].

ApoE is predominantly synthesized in the liver and in blood and is lipidated. ApoE serves as the primary ligand for receptor-mediated clearance for triglyceride-depleted post-hydrolysed remnants of VLDL and chylomicrons [54] (Fig. 1). However, the *APOE ε4* allele is associated with a distributional shift of ApoE E4 to lipoproteins richer in triglyceride, interfering in lipolysis and delaying catabolism and clearance from blood of the lipoprotein moiety [55–58]. We contend that it is a reasonable proposition to suggest that an ApoE E4-mediated delay in metabolism and clearance of TRL remnant-Aβ may exacerbate age-associated microvascular sequale that lead to capillary breakdown and neurovascular inflammation (Fig. 2). Several recent studies support this hypothesis. Montagne et al. reported that individuals bearing *APOE ε4* (with the ε3/ε4 or ε4/ε4 alleles) displayed breakdown of the BBB in the hippocampus and medial temporal lobe [59]. The finding was indicated in cognitively unimpaired *APOE ε4* carriers and preceded classical AD pathology of frank cerebral amyloid deposition but was more severe in those with cognitive impairment. Indirect evidence comes from Liu et al., who showed that mice genetically engineered for human ApoE E4 restricted exclusively to liver compromised synaptic plasticity and cognition by compromising the cerebrovasculature [60]. Moreover, the *APOE ε4* allele exacerbated amyloid brain pathology when cross-bred with amyloid transgenic mice. However, it remains to be determined whether heightened risk for AD associated with *APOE ε4* reflects specifically a lipoprotein-Aβ-induced corruption of the cerebral microvasculature.

The effects of *APOE ε4* genotype on modulating the metabolism of hepatically derived VLDL is mirrored in the catabolism of intestinally derived postprandial chylomicrons, because they share the same catabolic cascade (Fig. 1). Limited studies suggest postprandial amyloidemia may occur in *APOE ε4* versus *APOE ε3* subjects. Six hours after ingestion of a lipid-rich meal, *APOE ε4* had threefold greater abundance in blood of intestinally derived chylomicrons (indicated as Apo B48) and 1.5-fold greater abundance of hepatically derived Apo B100, consistent with previous reports of reduced clearance rates of post-hydrolysed remnants [61]. Interestingly, a similar postprandial triglyceride excursion was indicated for individuals with *APOE ε4* and *APOE ε3*, consistent with the proposition that Aβ may have been elevated as a consequence of decreased catabolic processes. Less clear is potential differential effects of the apo E-dependent interaction with brain parenchymal cellular matrices and uptake by glia.

## Conclusion

There is an accumulating body of evidence that microvascular disruption is the first pathological feature realized in AD. Loss of barrier function is associated with neurovascular inflammation and neurodegeneration. Less clear is what accelerates age-associated cerebral capillary breakdown. Several studies suggest that peripheral blood homeostasis of Aβ, particularly associated with lipoproteins rich in triglyceride, regulate capillary integrity. Exaggerated vascular exposure to lipoproteins-Aβ is associated with loss of tight junction proteins, brain parenchymal extravasation of plasma proteins including lipoprotein-Aβ, brain atrophy and cognitive impairment.

In preclinical studies, it has been shown that dietary SFA increase the plasma abundance of lipoprotein-Aβ concomitant with capillary dysfunction. Clinical studies showing a delayed catabolism of nascent triglyceride-rich lipoproteins, which principally chaperone soluble Aβ in blood, suggest potential synergistic amplification of amyloidemia in blood and, by extension, microvascular disruption. The collective body of evidence suggests that attenuating the flux of systemic lipoprotein amyloid, particulary the remnants of triglyceride-rich lipoproteins, may confer microvascular protection and reduce risk for AD. Given the substantial knowledge of modulating peripheral metabolism of lipoproteins by lifestyle changes, lipid-lowering and apoprotein-targeted pharmacotherapies, new opportunities to reduce risk for AD and slow progression may potentially be realized.

### Contemporary dietary recommendations to reduce Alzheimer’s disease risk in the context of peripheral metabolism of lipoprotein-Aβ

Authoratative reviews consistently recommend healthy diets such as Mediterranean style, featuring greater ingestion of polyunsaturated, monounsaturated and omega-3 fatty acids being associated with decreased inflammation, increased insulin sensitivity and brain-derived neurotrophic factor [62–67]. Preclinical studies would predict that a Mediterranean diet would result in decreased synthesis and secretion of lipoprotein-Aβ and better preservation of the neurovascular junction [31, 48, 68, 69]. Population studies investigating dementia and AD risk confirm the health benefits of a Mediterranean diet and conversely demonstrate that Western style diets richer in SFA promote neurovascular inflammation and suppress production of brain-derived neurotrophic factor (BDNF), an important molecule involved in learning and memory [70–74]. However, to date, population studies have not reported if chronic dietary behaviour influences peripheral lipoprotein-Aβ homeostasis and neurovascular integrity per se.

Excessive intake of carbohydrate, particularly high glycaemic index food commodities, is associated with dyslipidemia, as a consequence of increased lipogenesis and secretion of nascent TRL [75, 76]. Notionally, excessive carbohydrate intake may also be associated with exaggerated secretion into blood of lipoprotein-Aβ; however, this remains to be proven.

Adequate intake of micronutrients, polyphenols and antioxidants is associated with healthy ageing and is relative to dementia and AD risk [77–79]. The Mediterranean and other tailored diets such as DASH and MIND are associated with better cognitive functioning and slower cognitive decline [80–82]. However, there are a paucity of studies that can shed insight into impact of specific micronutrients on lipoprotein-Aβ metabolism and the cerebral microvasculature.

Collectively, there is an accumulating and strong body of evidence that adherence to brain-healthy diets can reduce risk for AD. However, whether the mechanisms include positive modulation of the lipoprotein-Aβ/capillary axis remains to be reported.

## Data Availability

Not applicable.

## Abbreviations

AD: Alzheimer’s disease
Aβ: Amyloid-beta
Apo B-48: Apolipoprotein B-48
*APOE ε2/3/4*: Apolipoprotein E (gene)
apoE E2/3/4: Apolipoprotein E (protein)
BBB: Blood-brain barrier
CSF: Cerebrospinal fluid
CMR: Chylomicron remnants
EC: Endothelial cell
HDL: High-density lipoprotein
IDL: Intermediate-density lipoprotein
LDLR: LDL receptor
L-sAβ: Lipoprotein-bound sAβ
LPL: Lipoprotein lipase
LDL: Low-density lipoprotein
LRP-1: Low-density lipoprotein receptor-related protein-1
MCM: Mature chylomicron
MCI: Mild cognitive impairment
NCM: Nascent chylomicrons
PET: Positron-emission tomography
RAGE: Receptor for advanced glycation end products
SFA: Saturated fatty acids
sAβ: Soluble-Aβ
TJ: Tight junction
TRL: Triglyceride-rich lipoprotein
UFA: Unsaturated fatty acids
VLDL: Very-low-density lipoprotein

## References

[1] (WHO) WHO. Dementia 2022 [cited 2022 6 April]. Available from: https://www.who.int/news-room/fact-sheets/detail/dementia.

[2] Xia X, Jiang Q, McDermott J, Han JJ (2018). Aging and Alzheimer's disease: comparison and associations from molecular to system level. Aging Cell.

[3] Cui MY, Lin Y, Sheng JY, Zhang X, Cui RJ (2018). Exercise intervention associated with cognitive improvement in Alzheimer's disease. Neural Plast.

[4] Morris JK, Vidoni ED, Johnson DK, Van Sciver A, Mahnken JD, Honea RA (2017). Aerobic exercise for Alzheimer's disease: a randomized controlled pilot trial. PLoS One.

[5] Cao GY, Li M, Han L, Tayie F, Yao SS, Huang Z (2019). Dietary fat intake and cognitive function among older populations: a systematic review and meta-analysis. J Prev Alzheimers Dis.

[6] Petersson SD, Philippou E (2016). Mediterranean diet, cognitive function, and dementia: a systematic review of the evidence. Adv Nutr.

[7] McGrattan AM, McGuinness B, McKinley MC, Kee F, Passmore P, Woodside JV (2019). Diet and inflammation in cognitive ageing and Alzheimer's disease. Curr Nutr Rep.

[8] Havekes R, Heckman PRA, Wams EJ, Stasiukonyte N, Meerlo P, Eisel ULM (2019). Alzheimer's disease pathogenesis: the role of disturbed sleep in attenuated brain plasticity and neurodegenerative processes. Cell Signal.

[9] Shokri-Kojori E, Wang GJ, Wiers CE, Demiral SB, Guo M, Kim SW (2018). β-Amyloid accumulation in the human brain after one night of sleep deprivation. Proc Natl Acad Sci U S A.

[10] Selkoe DJ, Hardy J (2016). The amyloid hypothesis of Alzheimer's disease at 25 years. EMBO Mol Med.

[11] Moreno-García A, Kun A, Calero O, Medina M, Calero M. An overview of the role of lipofuscin in age-related neurodegeneration. Front Neurosci. 2018;12:464. 10.3389/fnins.2018.00464 PMC6041410 30026686

[12] Lin Y, Shan P-Y, Jiang W-J, Sheng C, Ma L (2019). Subjective cognitive decline: preclinical manifestation of Alzheimer’s disease. Neurol Sci.

[13] Wang X, Huang W, Su L, Xing Y, Jessen F, Sun Y (2020). Neuroimaging advances regarding subjective cognitive decline in preclinical Alzheimer’s disease. Mol Neurodegener.

[14] Vergallo A, Mégret L, Lista S, Cavedo E, Zetterberg H, Blennow K (2019). Plasma amyloid β 40/42 ratio predicts cerebral amyloidosis in cognitively normal individuals at risk for Alzheimer's disease. Alzheimers Dement.

[15] Nakamura A, Kaneko N, Villemagne VL, Kato T, Doecke J, Dore V (2018). High performance plasma amyloid-beta biomarkers for Alzheimer's disease. Nature..

[16] Pereira JB, Janelidze S, Stomrud E, Palmqvist S, van Westen D, Dage JL (2021). Plasma markers predict changes in amyloid, tau, atrophy and cognition in non-demented subjects. Brain..

[17] Doecke JD, Pérez-Grijalba V, Fandos N, Fowler C, Villemagne VL, Masters CL (2020). Total Aβ 42/Aβ 40 ratio in plasma predicts amyloid-PET status, independent of clinical AD diagnosis. Neurology..

[18] Schindler SE, Bollinger JG, Ovod V, Mawuenyega KG, Li Y, Gordon BA (2019). High-precision plasma β-amyloid 42/40 predicts current and future brain amyloidosis. Neurology..

[19] Rembach A, Watt AD, Wilson WJ, Villemagne VL, Burnham SC, Ellis KA (2014). Plasma amyloid-beta levels are significantly associated with a transition toward Alzheimer's disease as measured by cognitive decline and change in neocortical amyloid burden. J Alzheimers Dis.

[20] Fandos N, Pérez-Grijalba V, Pesini P, Olmos S, Bossa M, Villemagne VL (2017). Plasma amyloid β 42/40 ratios as biomarkers for amyloid β cerebral deposition in cognitively normal individuals. Alzheimer's Dement.

[21] Lam V, Takechi R, Hackett MJ, Francis R, Bynevelt M, Celliers LM (2021). Synthesis of human amyloid restricted to liver results in an Alzheimer disease–like neurodegenerative phenotype. PLoS Biol.

[22] Bosoi CR, Vandal M, Tournissac M, Leclerc M, Fanet H, Mitchell PL (2021). High-fat diet modulates hepatic amyloid β and cerebrosterol metabolism in the triple transgenic mouse model of Alzheimer’s disease. Hepatol Commun.

[23] Jin W-S, Bu X-L, Liu Y-H, Shen L-L, Zhuang Z-Q, Jiao S-S (2017). Plasma amyloid-beta levels in patients with different types of cancer. Neurotox Res.

[24] Maarouf CL, Walker JE, Sue LI, Dugger BN, Beach TG, Serrano GE (2018). Impaired hepatic amyloid-beta degradation in Alzheimer’s disease. PLoS One.

[25] Song Q, Huang M, Yao L, Wang X, Gu X, Chen J (2014). Lipoprotein-based nanoparticles rescue the memory loss of mice with Alzheimer’s disease by accelerating the clearance of amyloid-beta. ACS Nano.

[26] Biere AL, Ostaszewski B, Stimson ER, Hyman BT, Maggio JE, Selkoe DJ (1996). Amyloid beta-peptide is transported on lipoproteins and albumin in human plasma. J Biol Chem.

[27] Koudinov AR, Berezov TT, Kumar A, Koudinova NV (1998). Alzheimer's amyloid beta interaction with normal human plasma high density lipoprotein: association with apolipoprotein and lipids. Clin Chim Acta.

[28] Koudinov A, Matsubara E, Frangione B, Ghiso J (1994). The soluble form of Alzheimer′ s amyloid β protein is complexed to high density lipoprotein 3 and very high density lipoprotein in normal human plasma. Biochem Biophys Res Commun.

[29] Matsubara E, Sekijima Y, Tokuda T, Urakami K, Amari M, Shizuka-Ikeda M (2004). Soluble Abeta homeostasis in AD and DS: impairment of anti-amyloidogenic protection by lipoproteins. Neurobiol Aging.

[30] Galloway S, Takechi R, Pallebage-Gamarallage MM, Dhaliwal SS, Mamo JC (2009). Amyloid-beta colocalizes with apolipoprotein B in absorptive cells of the small intestine. Lipids Health Dis.

[31] Galloway S, Takechi R, Nesbit M, Pallebage-Gamarallage MM, Lam V, Mamo JCL (2019). The differential effects of fatty acids on enterocytic abundance of amyloid-beta. Lipids Health Dis.

[32] Lee YH, Martin JM, Maple RL, Tharp WG, Pratley RE (2009). Plasma amyloid-beta peptide levels correlate with adipocyte amyloid precursor protein gene expression in obese individuals. Neuroendocrinology..

[33] Lee YH, Tharp WG, Maple RL, Nair S, Permana PA, Pratley RE (2008). Amyloid precursor protein expression is upregulated in adipocytes in obesity. Obesity (Silver Spring).

[34] Mamo JC, Jian L, James AP, Flicker L, Esselmann H, Wiltfang J (2008). Plasma lipoprotein beta-amyloid in subjects with Alzheimer's disease or mild cognitive impairment. Ann Clin Biochem.

[35] Gu Y, Schupf N, Cosentino S, Luchsinger J, Scarmeas N (2012). Nutrient intake and plasma β-amyloid. Neurology..

[36] Subash S, Essa MM, Braidy N, Awlad-Thani K, Vaishnav R, Al-Adawi S (2015). Diet rich in date palm fruits improves memory, learning and reduces beta amyloid in transgenic mouse model of Alzheimer's disease. J Ayurveda Integr Med.

[37] Burgess BL, McIsaac SA, Naus KE, Chan JY, Tansley GH, Yang J (2006). Elevated plasma triglyceride levels precede amyloid deposition in Alzheimer’s disease mouse models with abundant Aβ in plasma. Neurobiol Dis.

[38] Muñoz Fernández SS, Lima Ribeiro SM (2018). Nutrition and Alzheimer disease. Clin Geriatr Med.

[39] Martins LB, Malheiros Silveira AL, Teixeira AL (2021). The link between nutrition and Alzheimer's disease: from prevention to treatment. Neurodegener Dis Manag.

[40] Romanenko M, Kholin V, Koliada A, Vaiserman A. Nutrition, gut microbiota, and Alzheimer's disease. Front Psychiatry. 2021;12:712673. 10.3389/fpsyt.2021.712673 PMC8374099 34421687

[41] Siri-Tarino PW, Sun Q, Hu FB, Krauss RM (2010). Meta-analysis of prospective cohort studies evaluating the association of saturated fat with cardiovascular disease. Am J Clin Nutr.

[42] Micha R, Mozaffarian D (2010). Saturated fat and cardiometabolic risk factors, coronary heart disease, stroke, and diabetes: a fresh look at the evidence. Lipids..

[43] Eskelinen MH, Ngandu T, Helkala EL, Tuomilehto J, Nissinen A, Soininen H (2008). Fat intake at midlife and cognitive impairment later in life: a population-based CAIDE study. Int J Geriatr Psychiatry.

[44] Ruan Y, Tang J, Guo X, Li K, Li D (2018). Dietary fat intake and risk of Alzheimer's disease and dementia: a meta-analysis of cohort studies. Curr Alzheimer Res.

[45] Laitinen MH, Ngandu T, Rovio S, Helkala EL, Uusitalo U, Viitanen M (2006). Fat intake at midlife and risk of dementia and Alzheimer's disease: a population-based study. Dement Geriatr Cogn Disord.

[46] Solfrizzi V, Colacicco AM, D'Introno A, Capurso C, Torres F, Rizzo C (2006). Dietary intake of unsaturated fatty acids and age-related cognitive decline: a 8.5-year follow-up of the Italian longitudinal study on aging. Neurobiol Aging.

[47] Zhu R-z, Chen M-q, Zhang Z-w, Wu T-y, Zhao W-H (2021). Dietary fatty acids and risk for Alzheimer's disease, dementia, and mild cognitive impairment: a prospective cohort meta-analysis. Nutrition..

[48] Takechi R, Galloway S, Pallebage-Gamarallage MM, Wellington CL, Johnsen RD, Dhaliwal SS (2010). Differential effects of dietary fatty acids on the cerebral distribution of plasma-derived apo B lipoproteins with amyloid-beta. Br J Nutr.

[49] Namba Y, Tsuchiya H, Ikeda K (1992). Apolipoprotein B immunoreactivity in senile plaque and vascular amyloids and neurofibrillary tangles in the brains of patients with Alzheimer's disease. Neurosci Lett.

[50] Strittmatter WJ, Roses AD (1996). Apolipoprotein E and Alzheimer's disease. Annu Rev Neurosci.

[51] Corder E, Saunders A, Strittmatter W, Schmechel D, Gaskell P, Small G (1993). Gene dose of apolipoprotein E type 4 allele and the risk of Alzheimer's disease in late onset families. Science..

[52] Sando SB, Melquist S, Cannon A, Hutton ML, Sletvold O, Saltvedt I (2008). APOE epsilon 4 lowers age at onset and is a high risk factor for Alzheimer's disease; a case control study from Central Norway. BMC Neurol.

[53] Angelopoulou E, Paudel YN, Papageorgiou SG, Piperi C (2021). APOE genotype and Alzheimer’s disease: the influence of lifestyle and environmental factors. ACS Chem Neurosci.

[54] Mahley RW (1988). Apolipoprotein E: cholesterol transport protein with expanding role in cell biology. Science (New York, NY).

[55] Saito H, Dhanasekaran P, Baldwin F, Weisgraber KH, Phillips MC, Lund-Katz S (2003). Effects of polymorphism on the lipid interaction of human apolipoprotein E. J Biol Chem.

[56] Campos E, Nakajima K, Tanaka A, Havel RJ (1992). Properties of an apolipoprotein E-enriched fraction of triglyceride-rich lipoproteins isolated from human blood plasma with a monoclonal antibody to apolipoprotein B-100. J Lipid Res.

[57] Weisgraber KH, Mahley RW, Kowal RC, Herz J, Goldstein JL, Brown MS (1990). Apolipoprotein C-I modulates the interaction of apolipoprotein E with beta-migrating very low density lipoproteins (beta-VLDL) and inhibits binding of beta-VLDL to low density lipoprotein receptor-related protein. J Biol Chem.

[58] Patrick CNR, Theo JCvB. (1996). Apolipoprotein E effectively inhibits lipoprotein lipase-mediated lipolysis of chylomicron-like triglyceride-rich lipid emulsions in vitro and in vivo. J Biol Chem.

[59] Montagne A, Nation D, Sagare A, Barisano G, Sweeney M, Chakhoyan A (2020). APOE4 leads to blood–brain barrier dysfunction predicting cognitive decline. Nature..

[60] Liu C-C, Zhao J, Fu Y, Inoue Y, Ren Y, Chen Y (2022). Peripheral apoE4 enhances Alzheimer’s pathology and impairs cognition by compromising cerebrovascular function. Nat Neurosci.

[61] Bergeron N, Havel RJ (1996). Prolonged postprandial responses of lipids and apolipoproteins in triglyceride-rich lipoproteins of individuals expressing an apolipoprotein epsilon 4 allele. J Clin Invest.

[62] Souza PR, Marques RM, Gomez EA, Colas RA, De Matteis R, Zak A (2020). Enriched marine oil supplements increase peripheral blood specialized pro-resolving mediators concentrations and reprogram host immune responses: a randomized double-blind placebo-controlled study. Circ Res.

[63] Ravaut G, Légiot A, Bergeron KF, Mounier C. Monounsaturated fatty acids in obesity-related inflammation. Int J Mol Sci. 2020;22(1):330. 10.3390/ijms22010330 PMC7795523 33396940

[64] Lee JS, Pinnamaneni SK, Eo SJ, Cho IH, Pyo JH, Kim CK (2006). Saturated, but not n-6 polyunsaturated, fatty acids induce insulin resistance: role of intramuscular accumulation of lipid metabolites. J Appl Physiol.

[65] Nardi F, Lipina C, Magill D, Hage Hassan R, Hajduch E, Gray A (2014). Enhanced insulin sensitivity associated with provision of mono and polyunsaturated fatty acids in skeletal muscle cells involves counter modulation of PP2A. PLoS One.

[66] Abdel-Maksoud SM, Hassanein SI, Gohar NA, Attia SMM, Gad MZ (2017). Investigation of brain-derived neurotrophic factor (BDNF) gene expression in hypothalamus of obese rats: modulation by omega-3 fatty acids. Nutr Neurosci.

[67] Pawełczyk T, Grancow-Grabka M, Trafalska E, Szemraj J, Żurner N, Pawełczyk A (2019). An increase in plasma brain derived neurotrophic factor levels is related to n-3 polyunsaturated fatty acid efficacy in first episode schizophrenia: secondary outcome analysis of the OFFER randomized clinical trial. Psychopharmacology.

[68] Lim GP, Calon F, Morihara T, Yang F, Teter B, Ubeda O (2005). A diet enriched with the omega-3 fatty acid docosahexaenoic acid reduces amyloid burden in an aged Alzheimer mouse model. J Neurosci.

[69] Qosa H, Mohamed LA, Batarseh YS, Alqahtani S, Ibrahim B, LeVine H (2015). Extra-virgin olive oil attenuates amyloid-β and tau pathologies in the brains of TgSwDI mice. J Nutr Biochem.

[70] Rainey-Smith SR, Gu Y, Gardener SL, Doecke JD, Villemagne VL, Brown BM (2018). Mediterranean diet adherence and rate of cerebral Aβ-amyloid accumulation: data from the Australian imaging, biomarkers and lifestyle study of ageing. Transl Psychiatry.

[71] Hoscheidt S, Sanderlin AH, Baker LD, Jung Y, Lockhart S, Kellar D (2022). Mediterranean and Western diet effects on Alzheimer's disease biomarkers, cerebral perfusion, and cognition in mid-life: a randomized trial. Alzheimers Dement.

[72] Sanchez-Villegas A, Galbete C, Martínez-González M, Alfredo M, Razquin C, Salas-Salvadó J (2011). The effect of the Mediterranean diet on plasma brain-derived neurotrophic factor (BDNF) levels: the PREDIMED-NAVARRA randomized trial. Nutr Neurosci.

[73] Wu A, Ying Z, Gomez-Pinilla F (2004). The interplay between oxidative stress and brain-derived neurotrophic factor modulates the outcome of a saturated fat diet on synaptic plasticity and cognition. Eur J Neurosci.

[74] Butler MJ, Cole RM, Deems NP, Belury MA, Barrientos RM (2020). Fatty food, fatty acids, and microglial priming in the adult and aged hippocampus and amygdala. Brain Behav Immun.

[75] Shin W-K, Shin S, Lee J-k, Kang D, Lee JE (2021). Carbohydrate intake and hyperlipidemia among population with high-carbohydrate diets: the health examinees gem study. Mol Nutr Food Res.

[76] Ma Y, Li Y, Chiriboga DE, Olendzki BC, Hebert JR, Li W (2006). Association between carbohydrate intake and serum lipids. J Am Coll Nutr.

[77] Richard MJ, Roussel AM (1999). Micronutrients and ageing: intakes and requirements. Proc Nutr Soc.

[78] Colizzi C (2019). The protective effects of polyphenols on Alzheimer's disease: a systematic review. Alzheimers Dement (N Y).

[79] Pritam P, Deka R, Bhardwaj A, Srivastava R, Kumar D, Jha AK (2022). Antioxidants in Alzheimer&rsquo;s disease: current therapeutic significance and future prospects. Biology..

[80] Morris MC, Tangney CC, Wang Y, Sacks FM, Barnes LL, Bennett DA (2015). MIND diet slows cognitive decline with aging. Alzheimers Dement.

[81] Psaltopoulou T, Sergentanis TN, Panagiotakos DB, Sergentanis IN, Kosti R, Scarmeas N (2013). Mediterranean diet, stroke, cognitive impairment, and depression: a meta-analysis. Ann Neurol.

[82] Tangney CC, Li H, Wang Y, Barnes L, Schneider JA, Bennett DA (2014). Relation of DASH- and Mediterranean-like dietary patterns to cognitive decline in older persons. Neurology..

